# Vaccination with novel low-molecular weight proteins secreted from *Trichinella spiralis* inhibits establishment of infection

**DOI:** 10.1371/journal.pntd.0008842

**Published:** 2020-11-18

**Authors:** Mellina T. Srey, Alessia Taccogna, Yelena Oksov, Sara Lustigman, Pei-Yi Tai, John Acord, Murray E. Selkirk, Tracey J. Lamb, David B. Guiliano

**Affiliations:** 1 Department of Pathology, University of Utah, Salt Lake City, Utah, United States of America; 2 School of Health, Sport and Bioscience, University of East London, London, United Kingdom; 3 Laboratory of Electron Microscopy, Lindsley F. Kimball Research Institute, New York Blood Center, New York, New York, United States of America; 4 Laboratory of Molecular Parasitology, Lindsley F. Kimball Research Institute, New York Blood Center, New York, New York, United States of America; 5 Cambridge Healthcare Research, London, United Kingdom; 6 Department of Life Sciences, Imperial College London, London, United Kingdom; George Washington University School of Medicine and Health Sciences, UNITED STATES

## Abstract

*Trichinella spiralis* muscle stage larvae (mL1) produce excretory-secreted products (ESPs), a complex mixture of protein, which are believed to be important for establishing or maintaining an infection niche within skeletal muscle and the intestine. Studies of both whole ESPs and individual cloned proteins have shown that some ESPs are potent immunogens capable of eliciting protective immune responses. Here we describe two novel proteins, **S**ecreted from **M**uscle stage **L**arvae SML-4 and SML-5 which are 15 kDa and 12 kDa respectively. The genes encoding these proteins are highly conserved within the Trichinellids, are constituents of mL1 ESP and localized in the parasite stichosome. While SML-5 is only expressed in mL1 and early stages of adult nematode development, SML-4 is a tyvosylated glycoprotein also produced by adult nematodes, indicating it may have a function in the enteral phase of the infection. Vaccination with these proteins resulted in an impaired establishment of adult stages and consequently a reduction in the burden of mL1 in BALB/c mice. This suggests that both proteins may be important for establishment of parasite infection of the intestine and are prophylactic vaccine candidates.

## Author summary

Author summary

Trichinellosis, also known as trichinosis, is a disease caused by consuming raw or undercooked meat from animals infected with muscle stage larvae of *Trichinella spiralis*. These worms cause infection when L1 (newborn) larvae migrate and invade skeletal muscle remodeling muscle cells into long-lived nurse cells which serve as reservoirs for the parasite. The establishment of nurse cells allows *T*. *spiralis* to better survive within the host because factors, such as blood vessel formation, bring required components for survival to the worms. We previously found three novel proteins termed **S**ecreted from **M**uscle stage **L**arvae (SML)-1, -2, and -3 by transcriptomic analysis. In this study, we further expand our previous findings by identifying two novel low molecular weight proteins SML-4 and SML-5, which can elicit protective responses against challenge infections with *T*. *spiralis*.

## Introduction

*Trichinella spiralis* is a parasitic nematode that can infect a wide variety of mammalian hosts, including humans. During *T*. *spiralis*’s life cycle this parasite occupies two intracellular niches within its host, skeletal muscle (muscle stage larvae, mL1) and intestinal epithelial cells (developing larvae, L2-adults). In these environments the parasite remodels the cells or tissues it inhabits. Skeletal muscle fibers are remodeled by newborn larvae (NBL) into a novel structure called a “nurse cell,” which serves as a long-term home as they develop into mL1[1]. After ingestion of mL1 by a new host, parasites invade intestinal epithelial cells and develop into adults [2–4]. The nematodes migrate through the epithelium, resulting in trails of dead cells. In these colonization processes the excretory-secreted products (ESPs) of the parasites are thought to drive invasion, migration, or the remodeling of host cells and tissues which support parasite development. The identification of these molecules and dissection of their functions continues to be the focus of numerous studies [5–8], especially with respect to better understanding its unique lifestyle. *T*. *spiralis* and other Trichinellids remain important human and agricultural pathogens with millions of people worldwide chronically infected, and are classified as a neglected tropical disease [9,10].

Infection of laboratory mice with *Trichinella* have helped define the processes and cells underlying the development of protective immunity to enteral and muscle stages of the parasite. Time course experiments have shown that parasite ESPs are rapidly recognized by the immune systems of rodents and both protein and glycan antigens can elicit protective immune response [11,12]. In addition, vaccination with ESPs can protect from subsequent challenge infections by promoting the development of both humoral and cellular responses to the enteral and muscle stages of the parasite [13,14]. Efforts to isolate individual protective constituents of the ESPs which can recapitulate the effects of whole ESP preparations have identified over a dozen candidate antigens, many of which are novel proteins with no clear homologues outside of other Trichinellids. This has made it difficult to further test or even speculate what their biological functions might be. The efficacy of ESP-based subunit vaccines has varied, but routinely it results in a 30–60% reduction in recovery of either adults and/or mL1 [14–27]. To date the most effective reported antigens have been within these studies and have included secreted proteins such as DNAse II, nudix hydrolase and serine or cysteine protease inhibitor homologues [12,13,18,19]. However, within each of these studies both antigen and vaccine formats (i.e. DNA, live delivery system, recombinant proteins) have varied widely with no clear patterns in what drives efficacy. Further study of the protective components of *T*. *spiralis* ESPs along with identification of the key determinants that influence their effectiveness in vaccination trials will provide a platform for building and refining general strategies for vaccination against *Trichinella* and other medically or agriculturally important nematode pathogens [28].

We have previously published the results of an informatics-based screen of the *T*. *spiralis* transcriptome that identified three novel proteins secreted by mL1 [6]. Here, we extend this study and describe two novel abundant transcripts encoding low molecular weight secreted proteins of unknown function. We provide an initial analysis of their biochemical characteristics, localization within larval and adult stichocytes, and show that both proteins provide high levels of protection in vaccinated mice against *T*. *spiralis* challenge infections. Based on these initial proof of principle results and parallel studies in other parasitic nematodes, we suggest that these two novel *T*. *spiralis* ESP proteins are key vaccine antigens worth exploring in vaccine development programs.

## Materials and methods

### Ethics statement

The study was approved by the Imperial College Ethical Review Committee and performed under license from the UK Home Office (PPL: 70/6073) and the University of Utah Institutional Animal Care Use Committee (Protocol Number 17–04002). All procedures were carried out in accordance with institutional guidelines for the care and use of animals.

### Parasite material

Mice and rats were purchased from Harlan (Oxford, UK) or Jackson Laboratories (Bar Harbor, Maine, USA). Food and water were available *ad libitum*. mL1 of *T*. *spiralis* (T1 ISS930) were recovered from chronically infected Sprague–Dawley rats (2 months post-infection or more). Larvae were isolated and ESPs collected after culturing in serum-free RPMI-1640 for 72–80 hours as described in Gounaris *et al*. [29]. Adult nematodes were isolated from infected rat intestines 2- or 6-days post-infection by sedimentation using a Baermann funnel. Day 6 adults were cultured for 3 days in serum-free RPMI-1640 and ESPs were collected as previously published [29]. NBL were separated from adult worms by sedimentation and immediately frozen for subsequent analysis.

Soluble whole worm extracts (SXT) were prepared by disruption of nematodes first in a custom-made Bessman tissue pulverizer and protein extraction buffer (25mM HEPES pH 7.5, 1.5% *n*-octyl β-D-glucopyranoside and SigmaFast Protease Inhibitor cocktail). Parasite soluble protein extracts were then extracted after 30 minutes incubation on ice, followed by brief sonication (90 sec, low power) and centrifugation at 15,000 x g for 15 minutes; SXT were stored at -20°C. The protein content of concentrated ESPs and SXTs was determined by the BCA (Pierce) or Bradford (BioRad) microplate assay.

### Identification, sequence confirmation and expression analysis of SMLs -4 and -5

The informatic procedures used to identify SML-4 and SML-5 have been previously published in Guiliano *et al*. [6]. Briefly, assemblies of publicly available clustered *T*. *spiralis* shotgun cDNA (EST) datasets were locally searched for open reading frames predicted to encode secreted proteins [30]. cDNA clusters identified to encode potential secreted proteins were then further analyzed, and those predominantly composed of clones from mL1 further analyzed.

For each candidate protein, a corresponding full-length cDNA sequence was identified in GenBank: SML-4, ps46d08.y1 and SML-5, ps03c05 (GenBank accession numbers BG521264.1 and BG520988.1). cDNA clones were obtained from Washington University (St. Lewis, Missouri, USA) using the nematode cDNA/EST clone provision service, and the sequences confirmed using vector and gene-specific primers. The verified sequences were deposited in GenBank (SML-4: MN755765, and SML-5: MN755766). SML-4 and SML-5 gene family members from other Trichinellids were identified within the assembled Trichinellid genome sequences deposited in Genbank using BLAST [31]. When required, *de novo* gene prediction was performed using Artemis and BLAST sequence alignments [32]. Multiple sequence alignment and phylogenetic analysis of SML-4 and SML-5 protein families was performed using ClustalX and PAUP [33,34]. For the phylogenetic analysis of the SML-4 and SML-5 protein datasets, a heuristic maximum parsimony (MP) search was performed using stepwise addition of taxa. A consensus tree was built from the trees saved during each search and the tree was tested by bootstrap analysis (10,000 replicates, using a heuristic algorithm). The expression of SML-4 and SML-5 at different *T*. *spiralis* life cycle stages was analyzed by reverse transcriptase (RT)-PCR. *T*. *spiralis* nematodes were flash frozen in Trizol (Invitrogen) and disrupted using a custom made Bessman tissue pulverizer and RNA isolated according to the manufacturer’s protocols. Isolated RNA was treated with RNase-free DNAse and then further purified using RNAeasy mini column purification (Qiagen). Reverse transcription was performed using 2μg of total RNA, Superscript II and oligo (dT) (Invitrogen) according to the manufacturer’s protocols. PCR was performed with gene specific SML primers (03c05.pET29b.F1: CATATGGATCAATCAGTATTTAAT, 03c05.pET29b.R1 CTCGAGTGCACAGACGATGAAATT, 46d08.pET29b.F1: CATATGGTAAAAATAATACCACGT, 46d08.pET29b.R1 CTCGAGATCAGCTTCTAATAGGTC), standard reaction conditions and PCR cycle parameters (one cycle 95°C for 3 min; 35 cycles at 94°C for 30 s, 55°C for 30 s, 72°C for 1 min; one cycle 72°C for 10 min) and Taq polymerase (NEB). PCR reactions were resolved on 2% agarose gels, visualized with ethidium bromide and documented. *T*. *spiralis* alpha tubulin was used as an internal positive control (GenBank sequence EU867518; Ts-TBA-1.F1: GGTCACATATGCACCGGTGAT, Ts-TBA-1.R1 GCTGTTGTATTAGATAACATGC), and reactions with no reverse transcriptase were used as negative controls.

### Bacterial expression and purification of SML proteins

Open reading frames corresponding to predicted mature proteins of SML-4 (amino acids 23–140) and SML-5 (amino acids 21–109) were fused to a poly-histidine epitope tag (His-tag) by cloning into the *Nde*I and *Xho*I sites of the *Escherichia coli* expression vector pET-29b (Novagen). Proteins were expressed using either standard IPTG induction or via culture in Overnight Express media (Merck Chemicals). Insoluble recombinant proteins were purified under denaturing conditions using nickel affinity chromatography and His-Bind Quick 900 cartridges (Merck Chemicals) or Ni-Sepharose FF using an FPLC system. Denaturing agents were then removed by dialysis or on-column refolding performed on an FPLC using Sephacryl S200-HR resin and a redox-refolding buffer. Proteins were buffer exchanged into PBS, concentrated and then quantified by the BCA or Bradford assay. SDS-PAGE and Coomassie blue staining was used to assess the purity of recombinant proteins. Endotoxin was removed from protein samples using Pierce High Capacity Endotoxin Removal Columns (Thermo Scientific). Following endotoxin removal, the Pierce Chromogenic Endotoxin Quant Kit (Thermo Scientific) was used to determine the success of endotoxin removal.

### Production of murine polyclonal antiserum and vaccination of animals with recombinant SML-4 and SML-5

Polyclonal antisera to SML-4 and SML-5 for use in Western blots, IHC and immunogold transmission electron microscopy (TEM) was produced in BALB/c mice. Antigens were emulsified in 1:1 ratio of Freund’s incomplete adjuvant (FICA) to a final concentration of 1mg/mL by light sonication. Mice were given a primary immunization 50μg of antigen and FICA via intraperitoneal injection. After four weeks animals were boosted twice, three weeks apart, with each boost containing 25μg of recombinant SML-4 or 5 in FICA. Antibody responses to SMLs were quantified by ELISA and Western blotting prior to final bleed and collection of serum.

For vaccination against SMLs BALB/c mice were given a primary immunization of 100μg of recombinant SML-4 or SML-5 absorbed onto 1mg Imject Alum (Sigma-Aldrich) via intraperitoneal injection. Animals were rested for three to four weeks and then boosted twice with 25μg of recombinant SML-4 or SML-5 in alum allowing a one-to-three-week interval between boosts. Unvaccinated animals were given intraperitoneal injections of PBS in alum following to the same schedule as vaccinated animals.

### SDS-PAGE and western blotting

Protein samples were resolved by 12 or 15% SDS-PAGE. For Western blot analysis, 0.5μg of recombinant or 10μg of ESP or soluble crude extract (SXT) protein samples were resolved by SDS-PAGE, transferred onto polyvinylidene fluoride (PVDF) membranes, and blocked with 5% skimmed milk powder/PBS/0.1% Tween-20 for 1 hour. Blots were then incubated at 4°C overnight with anti-SML-4 or anti-SML-5 murine polyclonal antisera diluted (1:200) in blocking solution and pre-absorbed with *E*. *coli* lysate (Promega). Antibody binding was visualized using standard procedures with horseradish peroxidase (HRP)-conjugated anti-mouse IgG secondary antibody (BioRad) and chemiluminescent detection (ECL, Amersham).

Deglycosylation of ESP using N-Glycosidase F (PNGase F, NEB) was performed according to the manufacturer’s protocol. Briefly, 10μg of crude protein extract was denatured, 50 U ml^-1^ of enzyme added and the reaction incubated for 12 hours at 37°C. PNGase F reactions were resolved by SDS-PAGE and visualized by Western blotting using SML-4 or SML-5 specific antisera.

The antigenicity of the *E*. *coli*-expressed recombinant SML-4 and SML-5 was examined by Western blot using pooled sera collected from chronically infected BALB/c mice. Antibody binding was detected using goat anti-mouse IgG-HRP (clone 1030–05, Southern Biotech).

### Cellular localization of native SMLs

Muscles infected with *T*. *spiralis* were collected from rats 40 days post-infection. Tissue or isolated mL1 were fixed in 10% neutral buffered formalin overnight and then embedded in paraffin according to standard protocols. Paraffin sections were de-waxed with Histoclear, rehydrated, and antigen retrieval was performed by microwaving sections in a solution of citrate-based antigen unmasking solution (Vector Labs) for 15 minutes. Sections were incubated in 2% H_2_O_2_ to remove endogenous peroxidase activity and then blocked in PBS/5% normal goat serum/5% BSA for 1 hour at room temperature. Endogenous biotin and streptavidin binding sites were blocked using a commercial blocking kit (Vector Labs). Sections were incubated overnight at 4°C with murine anti-SML-4 or anti-SML-5 (diluted 1:100–1:200) in blocking solution, washed thoroughly with wash buffer, incubated with biotin-conjugated goat anti-mouse IgG (Jackson ImmunoResearch Labs) for 1 hour, washed again and detected with Streptavidin-HRP complex and DAB substrate (3,3'- diaminobenzidine, Vector Labs). Sections were counterstained with Mayer’s hematoxylin, mounted with DPX mounting media (BDH), and visualized by light microscopy.

For immunogold TEM, adult *T*. *spiralis* were fixed for 1 hour in 0.25% glutaraldehyde and 1% sucrose in 0.1M phosphate buffer pH 7.4 and then processed for immunoelectron microscopy as previously described [35,36]. mL1 were fixed in 4% paraformaldehyde overnight and then processed for TEM in an identical fashion to adult *T*. *spiralis*. For immuno-localization of the native parasite protein corresponding to recombinant SML-4 and SML-5, thin sections (70 nm) of embedded worms were incubated with anti-SML-4 and anti-SML-5 murine polyclonal antisera (1:20 dilution), followed by incubation with 15 nm gold particles coated with goat anti-mouse IgG (Amersham Life Sciences, Piscataway, NJ). Pre-immune serum was used as the control.

### ELISA analysis of sera collected from animals vaccinated with recombinant SML-4 and SML-5

ELISAs for recombinant SML-4 or SML-5 proteins were performed using 96 well plates (Nunc Polysorp) coated with 50μL of 0.05 M carbonate buffer containing 5μg/mL of recombinant SML-4 or SML-5. The plates were incubated at 4°C overnight. Plates were blocked with 1% BSA or 5% non-fat milk and sera samples were loaded at a dilution of 1:200 or 1:1000. After an overnight incubation, 50μL of anti-mouse detection antibody conjugated to horseradish peroxidase was added according to the manufacturer’s instructions (all Southern Biotech). Bound antibodies were detected using 100μL/well of 2,2'-azino-bis-(3-ethylbenzthiazoline-6-sulfonic acid) Super AquaBlue ELISA substrate (Invitrogen) and readings were taken at a wavelength of 405 nm.

### Cellular responses in splenocytes from mice vaccinated with recombinant SML-4 and SML-5

Spleens were removed from vaccinated animals one week after the second boost, and splenocytes isolated aseptically using standard methods. 1 X 10^6^ splenocytes per well were stimulated in 96 well round bottom plates for 72 hours with 20 μg endotoxin-free recombinant SML-4 or SML-5 protein. Concanavalin A (MP Biomedicals) was added at a concentration of 1μg/mL as a positive control stimulus, and media alone served as a negative control. Supernatants were analyzed by Luminex (ProcartaPlex kits; ThermoFisher) according to the manufacturer’s instructions. After 72 hours, splenocytes were transferred to a fresh 96 well flat-bottomed plate containing bound α-CD3 (clone 17A2, Biolegend) and cells were restimulated for 6 hours along with soluble α-CD28 (clone 37.51, Biolegend) with the addition of Brefeldin A (Biolegend) in the last 4 hours of stimulation. After blocking with addition of 10 μg/mL CD16/32 (clone 93, Biolegend), cells were then surface stained for CD4 (PerCP-Cy5.5, clone GK1.5, Biolegend), CD44 (Alexa Fluor 700, clone IM7, eBioscience) and zombie live/dead (BV 510, Biolegend). After fixation and permeabilization using Fix/Perm buffer (BD) cells were intracellularly stained for IFN-γ (PE, clone XMG1.2, Biolegend) and IL-4 (Pe-Cy7, clone BVD6-24G2, eBioscience). Cells were read on an LSRFortessa X-20 flow cytometer (BD) and data analyzed using FlowJo software.

### Vaccination and challenge of BALB/c mice immunized with recombinant SML-4 and SML-5

Groups of 8–10 female BALB/c mice 4–6 weeks old were purchased from Harlan (Oxford, UK) or Jackson Laboratories (Bar Harbor, Maine, USA) and immunized as described above. Pre-vaccination control sera were collected prior to vaccination. Control animals were immunized with PBS and alum.

Immunogenicity of the vaccines was checked by ELISA one week after the second boost or by western blot before mice were challenged with either 250 (moderate dose) or 500 *T*. *spiralis* (high dose) mL1. Forty days post-infection serum was collected from infected animals and the number of encysted mL1 recovered from each animal quantified after digestion of mouse carcasses to assess the effect of vaccination on expulsion of adult worms. This was further verified by enumerating the recovery of adult worms from the intestines of BALB/c mice infected with 500 *T*. *spiralis* (high dose) mL1 at days 2, 6, 9, and 12 post-infection. Briefly, the intestine was opened lengthwise and rinsed with PBS and adults were collected by sedimentation using a Baermann funnel before being counted.

### Statistical analysis

All experiments, with the exception of adult recoveries over 4 different time points and high-dose challenge (Fig 7), were undertaken at least twice. ELISA and Luminex data were analyzed using GraphPad Prism 8.0 software. One-tailed Mann-Whitney U tests were performed in Prism to test for production of antibodies and cytokines relative to negative controls, and p values < 0.05 were considered statistically significant. Flow cytometry data was analyzed using FlowJo version 10.5.3. *T*. *spiralis* mL1 and adult worm recovery were analyzed using ANOVA or General Linear Modeling to remove the effects of experimental variation (Minitab Inc). Dunnett’s pairwise comparison post-test was used to determine differences between experimental groups. Where duplicate experiments were carried out, experimental consistency was evaluated by inclusion of a vaccination-by-experiment interaction term in the model and was not found to be statistically significant. Residual variation was analyzed for normality (Anderson Darling test) and homogeneity of variance (Levene’s test) in each case. The mL1 and IgG2a data were logarithmically transformed, and the adult recoveries (n+1) were square root transformed to meet the requirements of parametric testing.

## Results

### Identification and expression of SML-4 and SML-5

The candidate secreted proteins abundantly represented in the EST datasets isolated in the informatics-based analysis previously described by Guiliano *et al*. [6] are shown in S1 Table. SMLs 1–3 were identified and characterized in this initial analysis [6]. A number of other EST clusters of transcripts encoding proteins previously identified by proteomic analysis of the ESPs of *T*. *spiralis* mL1 were also identified and include gp45, gp43, Ts53, prosaposin, and GM2A [37].

**Fig 1:**
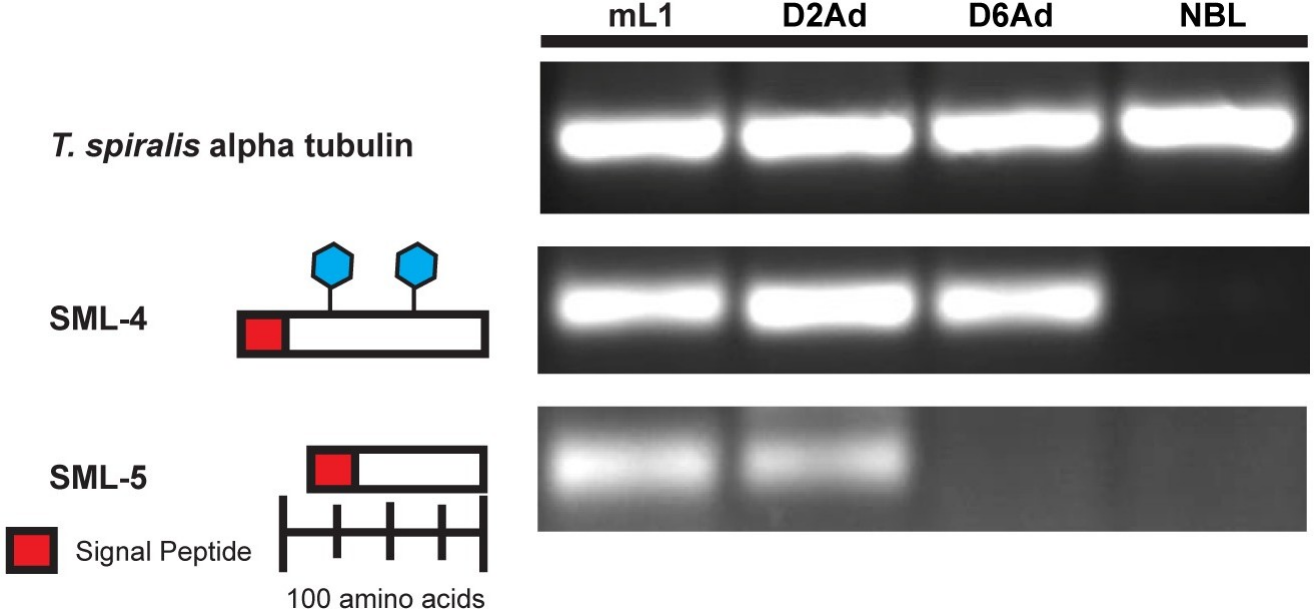
Bioinformatic and expression analysis of two *T*. *spiralis* abundant novel transcripts. The cartoon shows features identified in predicted protein translations of two abundant novel transcripts found within the *T*. *spiralis* mL1 EST dataset. These include potential signal peptides and confirmed N-linked glycosylation sites. RT-PCR showing the expression of these transcripts at different life cycle stages is also shown, with *T*. *spiralis* alpha-tubulin specific primers included to assess the quality of the cDNA used in the expression analysis. mL1: muscle stage larvae, D2Ad: day 2 adults D6Ad: day 6 adults, NBL: newborn larvae.

The candidates six and eleven in S1 Table are both are novel transcripts encoding low molecular weight proteins (15 kDa and 12 kDa), which we have designated SML-4 and SML-5, respectively. The predicted proteins of both are novel with no similarity to any sequences in GenBank, other than their cognizant segments of the recently sequenced *T*. *spiralis* genome [38]. Alignment and phylogenetic analysis of the SML-4 and SML-5 gene families revealed that both proteins are highly conserved within the genus and all identified genes had conserved gene structures and intron positions (Fig 1, S1 and S2 Figs). Analysis of the *Trichinella* T6 genome indicates that SML-4 is present as a multicopy gene family with three genes identified in the *Trichinella* T6 genomic contigs submitted to Genbank. Similarly, analysis of SML-5 indicates it is present as a multicopy gene family in *Trichinella nativa*. The predicted polypeptide of SML-4 contains two potential N-glycosylation sites (Fig 1) and was identified as a tyvosylated glycoprotein in the proteomic analysis of mL1 ESP published by Robinson and Connolly [37]. Analysis of SML-4 and SML-5 stage-specific transcript expression by RT-PCR revealed that mRNA transcripts for both genes were detectable in mL1 and day 2 post-infection in developing young adults. Transcript for SML-4 but not SML-5 was detectable in day 6 post-infection mature adults (Fig 1) while transcripts for both genes were not detected in NBL.

### Secretion and localization of SML-4 and SML-5

**Fig 2:**
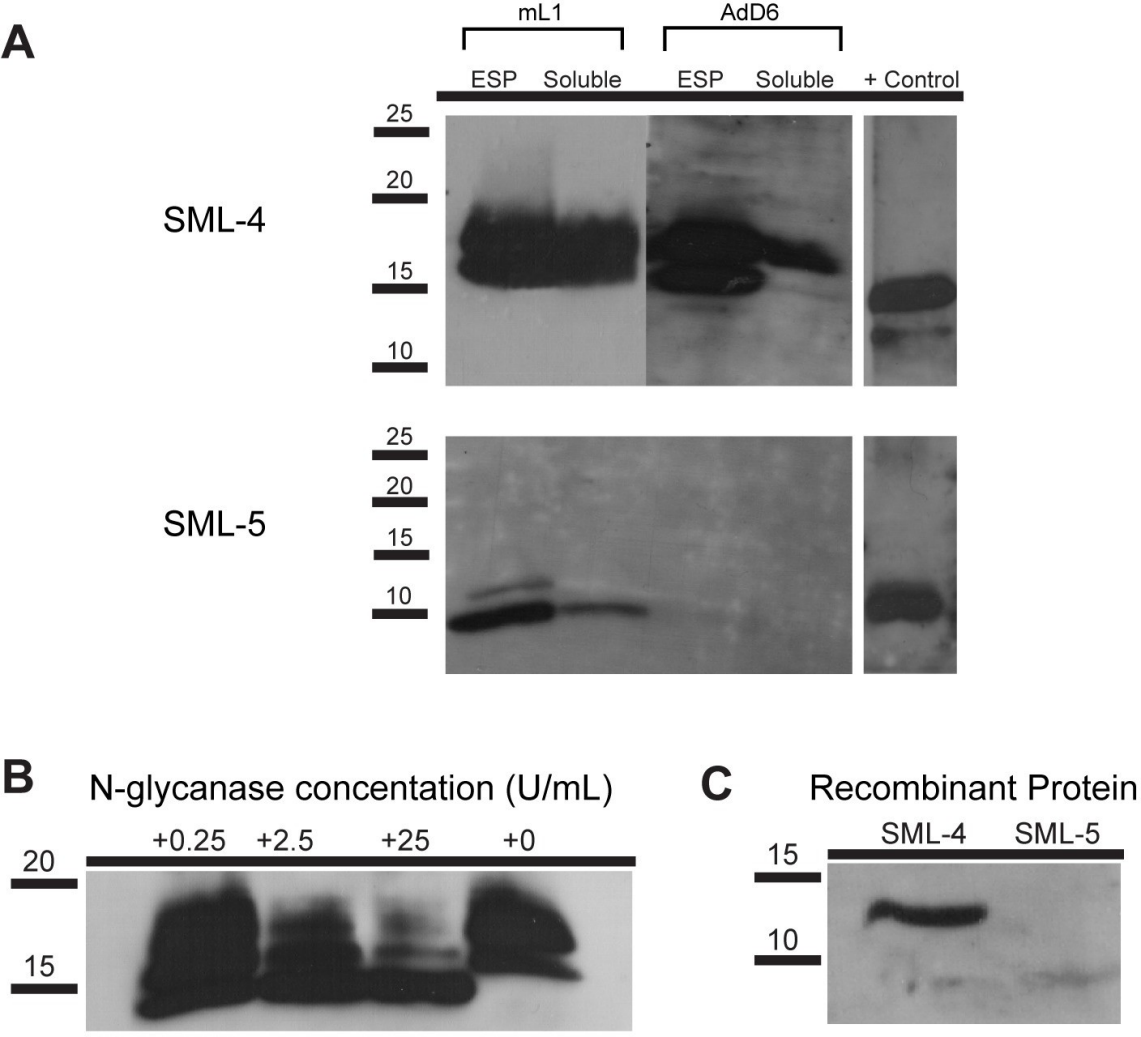
SMLs 4 and 5 are secreted by *T*. *spiralis*. (A) Western blot of *T*. *spiralis* excretory-secreted proteins (ESP), soluble crude extracts (Soluble, SXT) or recombinant protein (+) probed with murine polyclonal antisera to SML-4 or SML-5. (B) *T*. *spiralis* ESPs were treated with PNGase F over a range of enzyme concentrations (0.25–25 U/mL) and probed by western blot with antisera to SML-4. (C) Recombinant SML-4 and SML-5 proteins were probed by western blot with pooled BALB/c murine infection sera. Infection sera shows strong reactivity for SML-4 and weaker reactivity for SML-5. Molecular mass markers are shown in kilodaltons.

Recombinant SML-4 and SML-5 were expressed in *E*. *coli* as His-tag fusion proteins and purified using nickel affinity chromatography. Recombinant proteins were used to generate murine polyclonal antisera, which was then used as a probe in western blots with parasite soluble crude extracts (SXT) and excretory-secreted proteins (ESP). Murine antisera generated against SML-4 detected two protein species migrating at 16 and 18kDa (Fig 2A) in the ESP and SXT of mL1 and adult worms. SML-5 was identified in both mL1 ESP and SXT, with the corresponding native protein migrating at approximately 9kDa (Fig 2A). While we failed to detect SML-5 in our adult protein samples this does not preclude it being present at levels below the detection of the Western blots.

Treatment of mL1 ESP with PNGase F demonstrated that as increasing concentrations of enzyme were added the 16 and 18 kDa species progressively resolved into a single 14 kDa species. This indicates the two SML-4 protein species identified are glycosylated and likely represent distinct glycoforms (Fig 2B). The digestion pattern indicates that within these two species post-translational modification occurs at a single (16 kDa) or both (18 kDa) of the potential N-glycosylation sites. Western blot analysis using sera isolated from chronically infected BALB/c mice infected with *T*. *spiralis* demonstrated that naturally accruing IgG antibodies recognize recombinant SML-4, but not recombinant SML-5 (Fig 2C).

### Spatial localization of native SML-4 and SML-5

**Fig 3:**
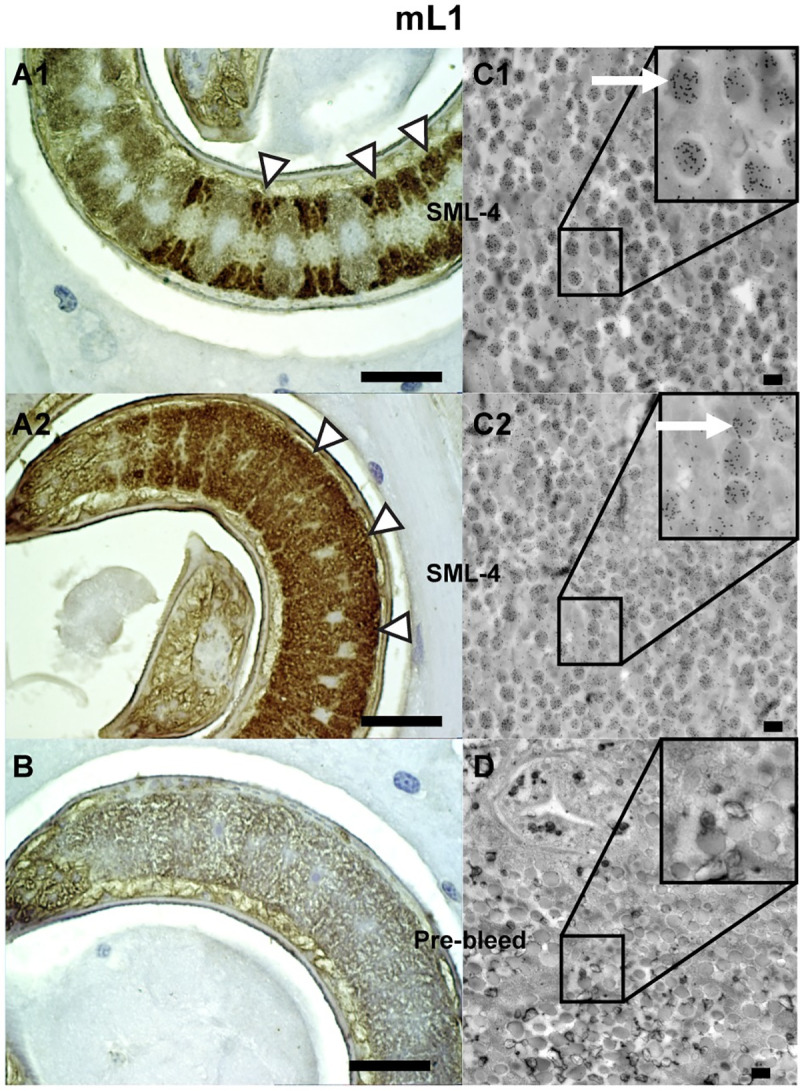
Histochemical and ultrastructural localization of SML-4. (A and B) Formalin-fixed paraffin embedded *T*. *spiralis*-infected rat tissue 28 days post-infection probed with anti-SML-4 (A1 and A2) or pre-vaccination control sera (B) and detected with HRP-conjugated anti-IgG and DAB substrate. Sections were counterstained with Mayer’s hematoxylin. The white arrows show areas of staining within the mL1 stichocytes. (C and D) TEM sections of *T*. *spiralis* mL1 were probed with anti-SML-4 (C1 and C2) or pre-vaccination control sera (D) and visualized with goat anti-mouse IgG coupled to 15 nm gold particles (black dots). Localization of SML-4 to granules within the mL1 β-stichocytes (white arrows) can be observed. No staining of α-stichocyte granules was observed. Black scale bars in twenty μm (A and B) and five hundred nm (C and D) are shown in each image.

**Fig 4:**
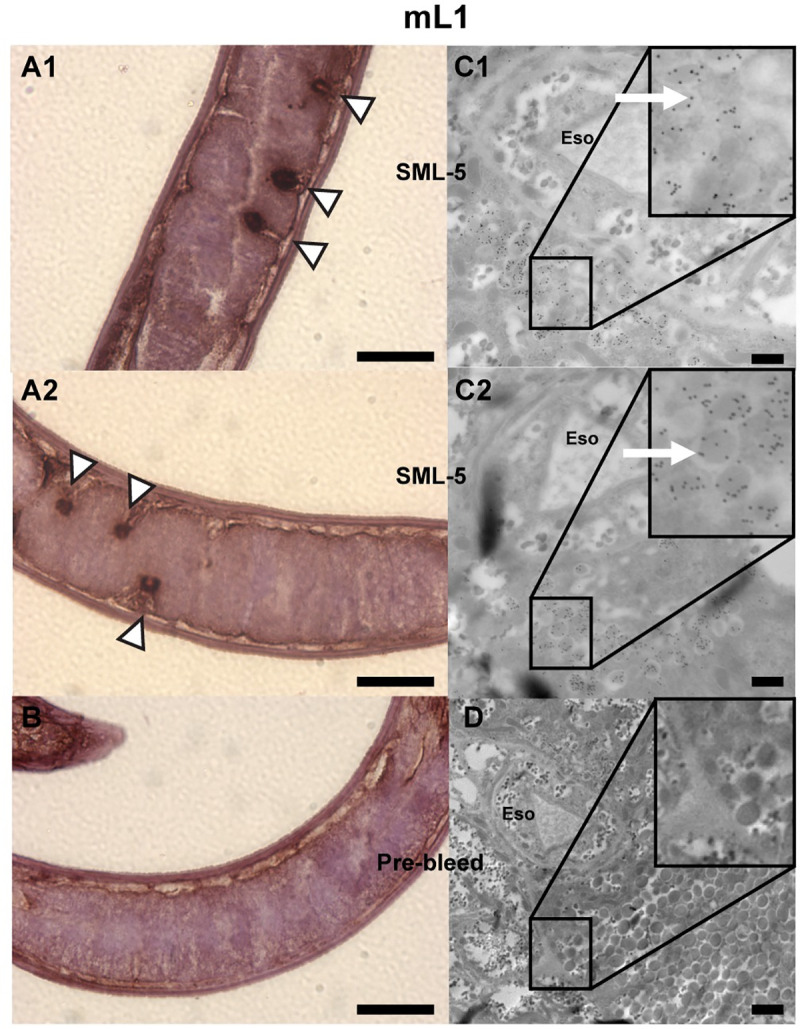
Histochemical and ultrastructural localization of SML-5. (A and B) Formalin-fixed paraffin embedded *T*. *spiralis* mL1 probed with anti-SML-5 (A1 and A2) or pre-vaccination control sera (B) and detected with HRP-conjugated anti-IgG and DAB substrate. Sections were counterstained with Mayer’s hematoxylin. The white arrows show areas of staining within specific regions of the mL1 α-stichocytes. (C and D) TEM sections of *T*. *spiralis* mL1 were probed with anti-SML-5 (C1 and C2) or pre-vaccination control sera (D) and visualized with goat anti-mouse IgG coupled to 15 nm gold particles (black dots). Localization of SML-5 to a subset of granules adjacent to esophagus can be observed. Eso: Esophagus. Black scale bars in twenty μm (A and B) and five hundred nm (C and D) are shown in each image.

**Fig 5:**
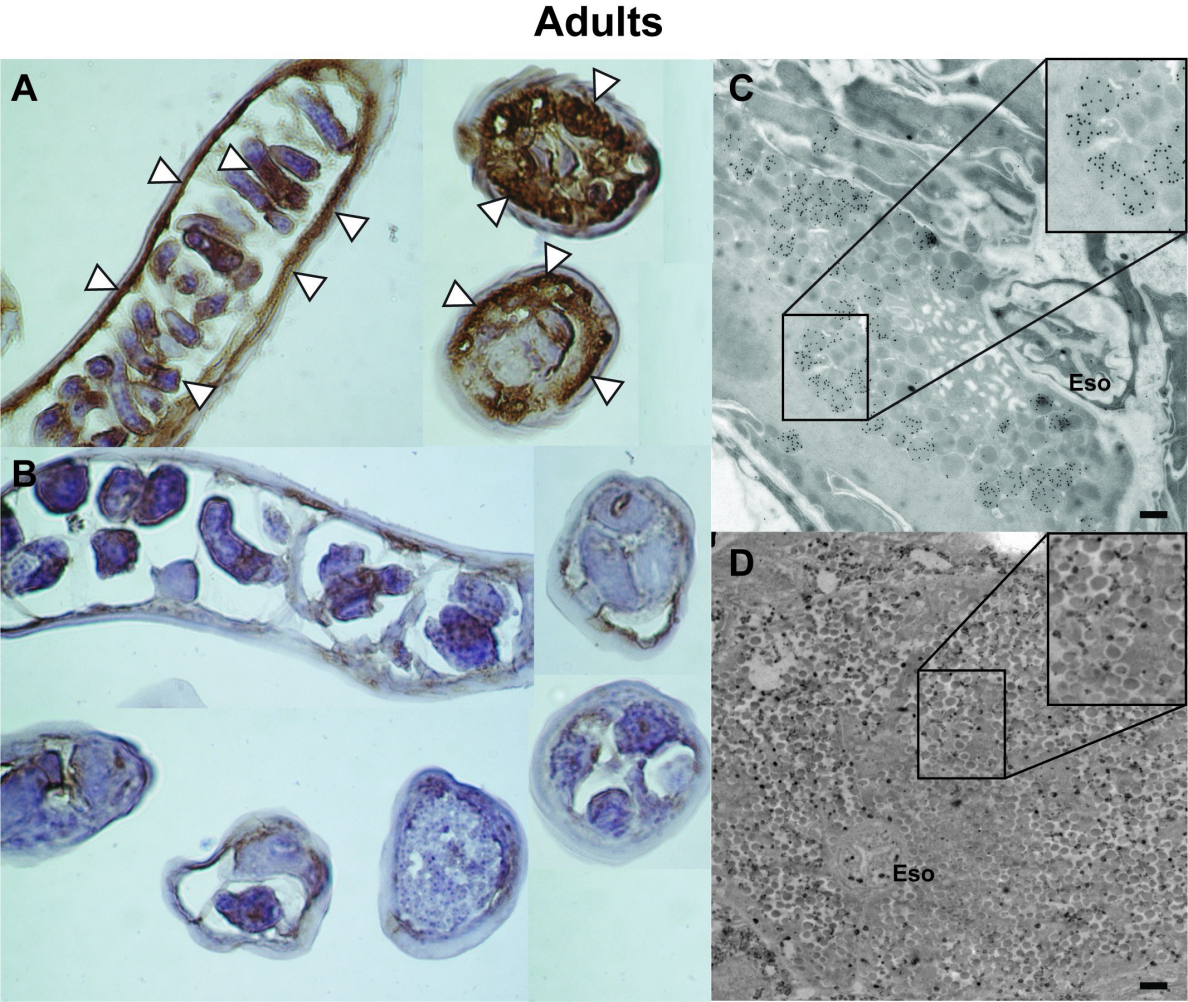
Histochemical and ultrastructural localization of SML-4 in adult nematodes. (A and B) Formalin-fixed paraffin embedded *T*. *spiralis* adults probed with anti-SML-4 (A) or pre-vaccination control sera (B) and detected with HRP-conjugated anti-IgG and DAB substrate. Sections were counterstained with Mayer’s hematoxylin. The white arrows show areas of staining within specific regions of the adult stichocytes and the uterine wall. (C and D) TEM sections of *T*. *spiralis* adults were probed with anti-SML-4 (C) or pre-vaccination control sera (D) and visualized with goat anti-mouse IgG coupled to 15 nm gold particles (black dots). Localization of SML-4 to granules adjacent to the esophagus can be observed. Eso: Esophagus. Black scale bars five hundred nm are shown in image C and D.

Two different techniques were used to localize the native SML-4 and SML-5 either within the nematodes or *in situ* within muscles of infected mice. Analysis of formalin-fixed/paraffin embedded tissue showed SML-4 is localized in the mL1 β-stichocytes within the stichosome (Fig 3A and 3B), and this was confirmed by immunogold TEM, which showed SML-4 is present in dark granules within the stichocytes (Fig 3C and 3D). In formalin-fixed/paraffin embedded tissue SML-5 was found to be localized in the α-stichocytes within the stichosome of the larvae (Fig 4A and 4B). Similar to SML-4, immunogold TEM localized SML-5 to the stichocytes in granules adjacent to the esophagus (Fig 4C and 4D). Assignment of localization of the SML proteins to either the α- or β-stichocytes was based on their position within the stichosome with the β-stichocytes being found towards the anterior portion of the stichosome and comprising the majority of the stichocyte cells. The α-stichocytes are the posterior ~10 stichocyte cells found in close proximity to the genital primordium of the larvae, as described by Despommier and Muller [39]. We were unable to localize either protein outside the parasite in nurse cells using both techniques. In adults, SML-4 was localized in the uterine walls of female nematodes and stichosomes (Fig 5A and 5B). This observation was confirmed by immunogold TEM where SML-4 was found to be localized in small granules adjacent to the esophagus (Fig 5C and 5D).

### Vaccination with SML-4 and SML-5 induces mixed Th1/Th2 responses

**Fig 6:**
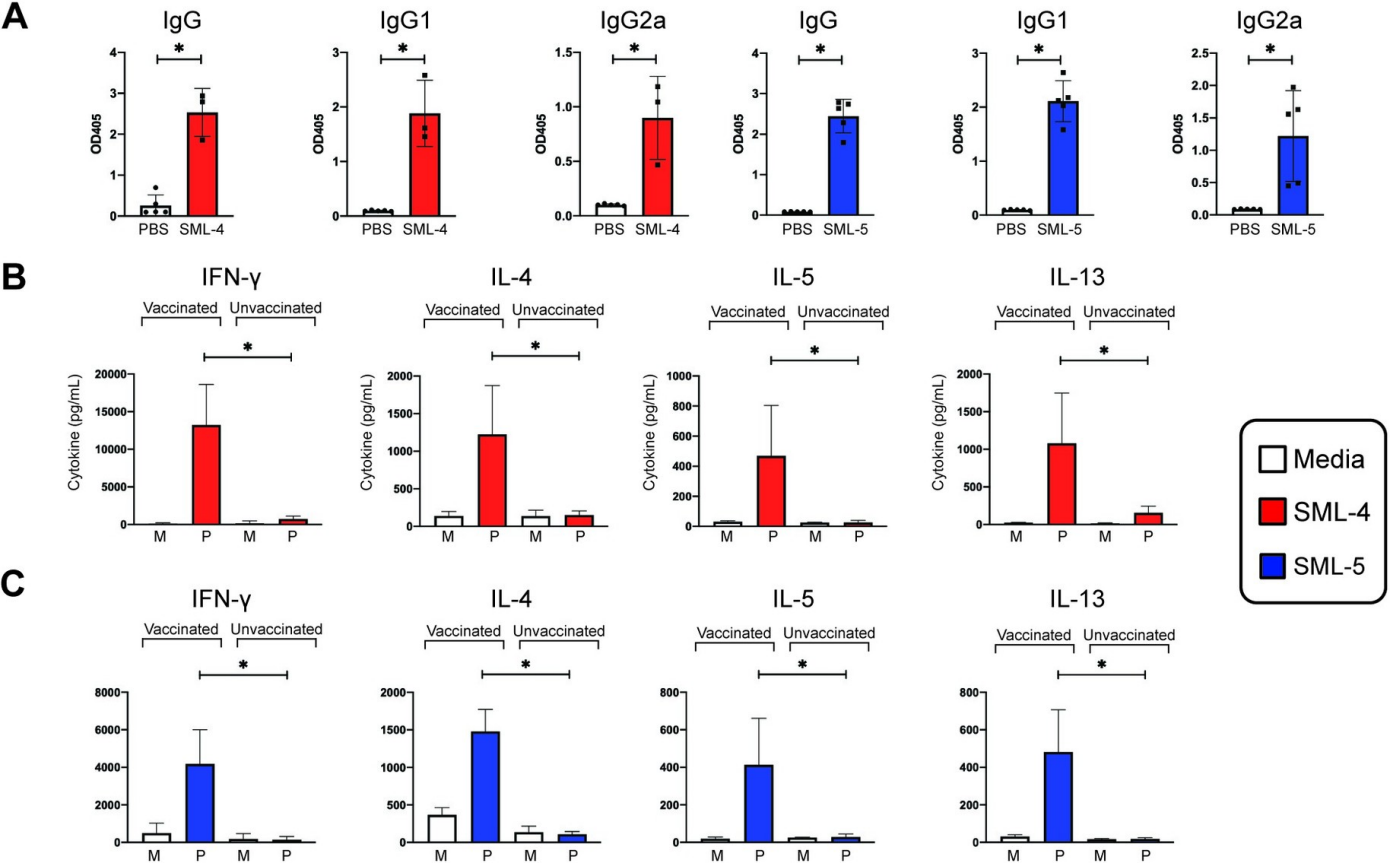
Parenteral vaccination with SML-4 or SML-5 in alum induces mixed Th1/Th2 cellular and humoral responses. (A) Recall responses of BALB/c mice immunized via intraperitoneal injection with SML-4 or SML-5 protein in alum adjuvant were analyzed via ELISA assays for IgG, IgG1 and IgG2a. Unvaccinated mice were given intraperitoneal injections of PBS in alum. (B and C) Supernatants from splenocytes stimulated for 72 hours with media, SML-4 (B), or SML-5 (C) were analyzed for cytokines via Luminex assays. “M” indicates stimulation with media, while “P” indicates stimulation with SML-4 or SML-5 protein. Figures are representative of two independent experiments with n = 4–5 per group.

Female BALB/c mice were vaccinated with recombinant SML-4 or SML-5 protein formulated with alum adjuvant. One week after the last immunization, antigen-specific antibodies were measured using ELISA. Immunization induced an IgG response to SML-4 and SML-5 (Fig 6A). The total IgG responses measured were composed of both IgG1 and IgG2a responses (one-tailed Mann-Whitney U test, SML-4 p = 0.0179; SML-5 p = 0.004), suggestive of a mixed Th1 and Th2 response in both vaccinations. Supernatants from splenocytes stimulated for 72 hours with SML-4 or SML-5 protein supported the antibody isotype analysis with increases in Th2 (IL-4, IL-5, IL-13) as well as Th1 (IFN-γ) cytokine production (all one-tailed Mann-Whitney U test, p<0.05). Expression of cytokines by T cells was determined by flow cytometry analysis showing the development of IFN-γ and IL-4 producing CD4+ T cells in the culture (S3 Fig). However, the recall response to SML-4 was more biased towards type 1 response (more IFN-γ) than that to SML-5 protein (one-tailed Mann-Whitney U test, p = 0.0179) (Fig 6B and 6C).

### Vaccination with SML-4 and SML-5 protects BALB/c mice from challenge with *T*. *spiralis* larvae

**Fig 7:**
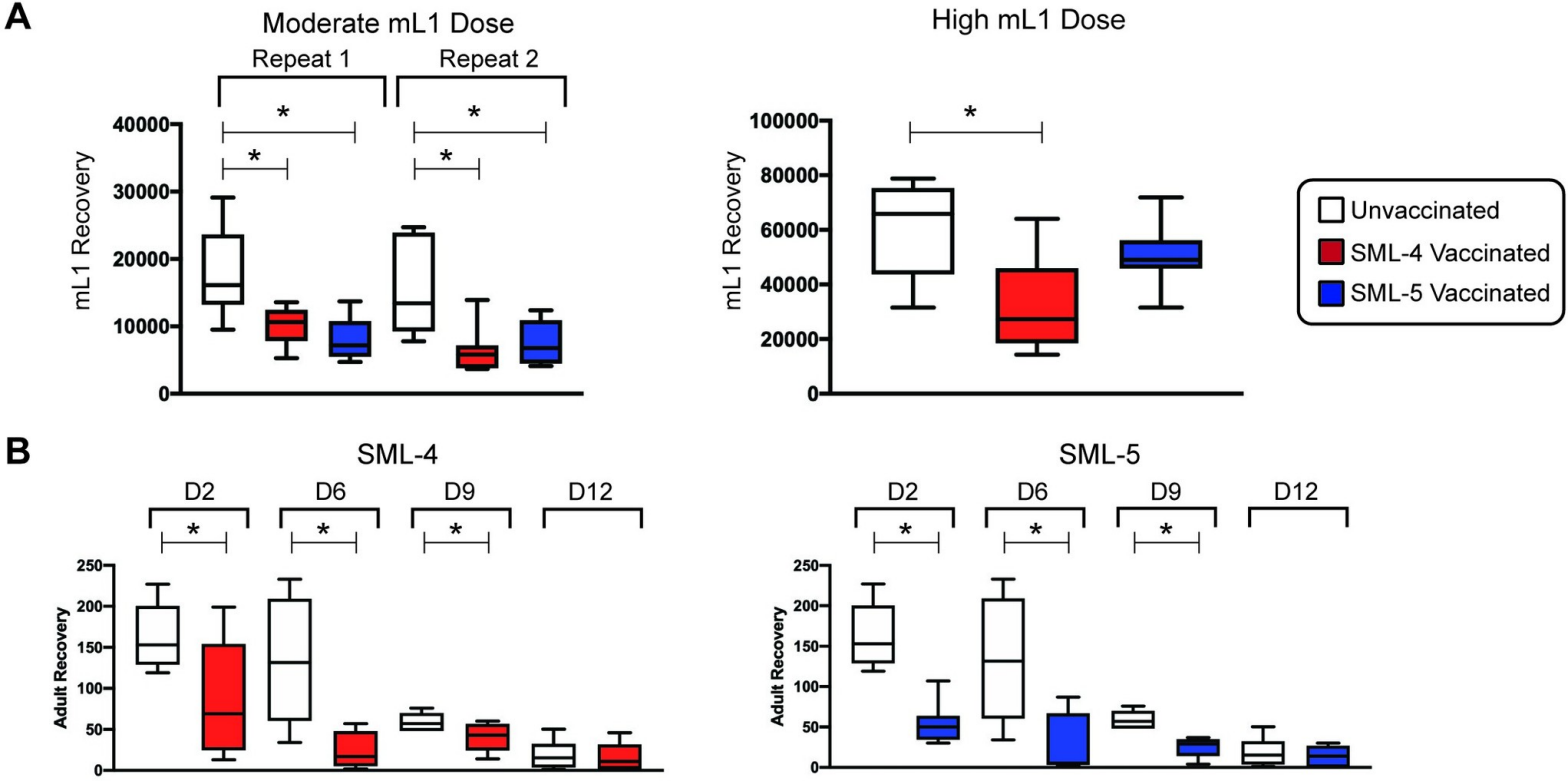
Vaccination with SML-4 and SML-5 is protective against *T*. *spiralis* infection. (A) BALB/c mice vaccinated with SML-4 or SML-5 protein, or unvaccinated control mice, were challenged with a moderate (250) or high (500) dose of infective mL1, and at forty days post-infection, the number of encysted mL1 collected from vaccinated mice was determined. (B) The number of adult worms recovered from vaccinated mice challenged with a moderate dose of infective mL1 was quantified at 2, 6, 9, and 12 days post-infection. Each experimental group contained 6–8 mice per time point for both A and B.

BALB/c mice vaccinated with SML-4 or SML-5 protein were challenged with a moderate (250) or high (500) dose of infective mL1. At forty days post-infection, the number of encysted mL1 collected from vaccinated mice challenged with a moderate dose was significantly lower compared to the control group (General Linear Modeling F_2,34_ = 13.24, p = 0.000; Dunnett’s post-test, p<0.05 for both antigens) (Fig 7A). There was a reduction in larvae recovered that ranged from 43 to 56% in both experiments in mice vaccinated with either protein compared to unvaccinated animals. Upon challenge of mice with a high dose of infectious larvae, vaccination with SML-4 was still protective with a 47% reduction in larvae recovered (one-way ANOVA F_2,19_ = 8.10, p = 0.003; Dunnett’s post-test, p<0.05), but the SML-5 vaccinated animals did not show a statistically significant difference in mL1 recovery as compared with control animals (Dunnett’s post-test, p>0.05). This indicates that SML-4 is a more effective vaccine candidate when compared to SML-5.

To determine if the reduced larval burden after vaccination with SML-4 or SML-5 is a result of reduced establishment of adult worms in the intestine, the adult worm recovery from groups of vaccinated mice that were challenged with a moderate dose of infective mL1 was quantified at 2, 6, 9, and 12 days post-infection (Fig 7B). Vaccination with either antigen accelerated the expulsion of adults over this time course of infection (General Linear Modeling F_2,75_ = 26.83, p = 0.000; Dunnett’s post-test, p<0.05 for both antigens). This protective effect was apparent from day 2 post-infection (one-way ANOVA F_2,21_ = 10.07; p = 0.000) showing a 67% and 48% reduction in adult recoveries for SML-4 or SML-5, respectively (Dunnett’s post-test, p<0.05 for both antigens). The efficacy of the single antigen vaccine is slightly lower than vaccinations with whole ESP which can reach 70–90% reductions in adult recovery but similar if not better than many previously published vaccines based on single recombinant protein proteins [13–16,18,20,21,23–25]. This suggests that the reduced recovery of mL1 for both vaccines was likely due to reduced establishment of adult worms early in the infection in vaccinated mice.

## Discussion

Analysis of ESTs from different lifecycle stages of the parasitic nematode *T*. *spiralis* has facilitated the identification of numerous new ESP proteins. Building on the previously published analysis of SML-1, -2 and, -3 [1] we now describe two novel low molecular weight ESPs SML-4 and SML-5. While these proteins are highly conserved across the Trichinellids, they do not show any homologies to proteins outside of the genus. Expression and localization of both proteins has been shown in the infectious larvae (SML-4 and SML-5) or adult stages (SML-4) of the parasite’s development. Immunohistochemical and immunogold localization of the SML-4 and SML-5 proteins indicates that while both are secreted via the stichocytes, they are produced in distinct populations of cells, with SML-4 localizing to β-stichocytes and SML-5 to α-stichocytes. Within adult worms, SML-4 is also localized to the reproductive structures of female nematodes, including the uterus. Comparison of stage-specific RT-PCR and the immunogold TEM data suggests that SML-4 is produced in adult females rather than NBLs. Biochemical analysis of native SML-4 has confirmed it is N-glycosylated at two sites; however, the presence of important Trichinellid glycans such as tyvelose remains to be assessed.

While it is difficult to speculate what the functions of these two proteins might be, their differential expression and localization suggests they may have roles linked to the development of mL1 into adults and/or the parasite’s transition into the enteral environment. The presence of SML-4 throughout this phase of the parasite’s lifecycle hints that its function is requisite for successful adaptation and persistence within the enteral niche. This contrasts with the restricted expression of SML-5 in the early stages of intestinal invasion, which could indicate that it might have a role more specific to the initial phases of infection or developmental processes, like molting, which occurs during the first two days of infection. While both SML-4 and SML-5 are constituents of mL1 ESP, protein localization studies do not indicate they are secreted into the nurse cell. However, given the specific limitations of these techniques, such as loss of antigenicity after fixation and low protein concentrations outside the nematode, we cannot exclude the possibility they are secreted into the nurse cell and participate in its development or maintenance.

Recombinant SML-4 and SML-5 can act as potent immunogens when administered to BALB/c mice with alum. Surprisingly, assessment of the cellular and humoral responses to these proteins indicates that despite being administered with alum, which promotes the development of Th2 responses, both proteins are capable of eliciting Th1 (SML-4) or mixed Th1/Th2 (SML-5) responses and mixed IgG Th1/Th2 associated isotype production. Preparations of recombinant SML proteins were routinely tested for LPS contamination prior to use (values < 0.6 ng endotoxin per mL) and low levels of residual endotoxin cannot fully explain the development of the Th1 or mixed Th1/Th2 responses we observe after immunization with these proteins. Potentially, these recombinant proteins have inherent polarizing Th1 properties which are skewing the development of balanced Th1/Th2 away from Th2 known to be activated in alum formulated vaccines. Such a potential bias has been observed with other nematode derived proteins [40] and may be linked to their functions at the host parasite interface and immune evasion.

Vaccination of BALB/c mice with these proteins prior to challenge infection has shown that both proteins are able to induce high levels of immune responses eliciting protective immunity. Assessment of infection time courses show that parasite attrition happens at early time points post-infection, indicating protective immune responses may be targeting the parasite during this critical point of adaptation and establishment in the enteral niche. In general vaccination generates adaptive immune responses against the target antigen(s) that can neutralize pathogens and pathogen-derived products. Although the majority of vaccinations are parenterally administered, immunity can extend to specific organs of the body. Given that SML-4 and SML-5 are secreted proteins from mL1, and that mL1 emerge into the small intestine which would have mucosal antibodies that recognize SML-4 and SML-5 from vaccination as measured in Fig 6, it is possible that the protective effects of vaccination arise from opsonization of secreted SML-4 and SML-5 preventing interaction with mucosal epithelial cells. As such modulation in environment by these proteins would not be possible. The mechanisms underlying this reduction in parasite numbers are yet to be defined; however, vaccination with these antigens did not cause any dramatic shifts in total anti-parasite IgG or IgG1 and IgG2a isotypes whose development have previously been linked to protective immunity after immunization with other *Trichinella* antigens.

Analysis of the *T*. *spiralis* transcriptome continues to yield novel proteins whose biological or immunological properties may be important in mediating evasive host-parasite interactions and/or the development of protective immune responses. Moving forward, while the biological functions of SML-4 and SML-5 remain unknown, we have established their efficacy as novel vaccinogens. Further work refining the antigenicity and mechanisms of action of these novel proteins will provide critical clues to design anti-Trichinosis vaccines with increased protective efficacy.

## Supporting information

S1 Fig Alignment and phylogenetic analysis of *T*. *spiralis* SML-4 and homologues identified in the genomes of other Trichinellids. (A) A multiple protein sequence alignment yielded by ClustalX analysis of the 16 SML-4 homologues found in sequenced genomes of Trichinellid species contained in Genbank. The Genbank accession number of the protein is given along with the species/isolate identifier. In five instances a manual genome prediction (MGP) was required to refine gene prediction models obtained from searching contigs isolated from the *T*. *nativa* (T2, JYDW01000232.1), *Trichinella* (T6, JYDK01000182.1), *T*. *pseudospiralis* (T4_3, JYDV01000046.1), *T*. *papuae* (T10, JYDO01000115.1) and *T*. *zimbabwensis* (T11, JYDP01000179.1) genomes. The location of the conserved intron site is indicated with a red triangle and potential N-glycosylation sites with the blue hexagons. (B) A rooted cladogram showing the consensus of the tree found in the maximum parsimony analysis of the SML-4 protein sequence alignment. Nodes which are supported bootstrap values of > 60% are shown and the bootstrap values are placed at the base of the node.
(TIF)

S2 Fig Alignment and phylogenetic analysis of *T*. *spiralis* SML-5 and homologues identified in the genomes of other Trichinellids. (A) A multiple protein sequence alignment yielded by ClustalX analysis of the 16 SML-5 homologues found in sequenced genomes of Trichinellid species contained in Genbank. The Genbank accession number of the protein is given along with the species/isolate identifier. The location of the conserved intron site is indicated with a red triangle. (B) A rooted cladogram showing the consensus of the tree found in the maximum parsimony analysis of the SML-5 protein sequence alignment. Nodes which are supported bootstrap values of > 60% are shown and the bootstrap values are placed at the base of the node.
(TIF)

S3 Fig Flow cytometry of activated CD4+ cells following recall responses in BALB/c mice vaccinated with SML-4 (A) or SML-5 (B) protein in alum adjuvant. Cell stimulations (ConA, SML-4, SML-5, or media) are indicated within respective flow plots. Figures are representative of two independent experiments with n = 5 per group.
(TIF)

S1 Table Candidates isolated from *T*. *spiralis* mL1 EST datasets. This table shows the top 11 candidates identified in the informatics screen of the *T*. *spiralis* mL1 EST dataset with their names and Genbank accession numbers. The columns next to the identifier show the number of ESTs for each transcript that were identified in the *T*. *spiralis* mL1 and adult EST datasets. Homologies to sequences contained within Genbank, the NEMBASE4 cluster (accessed 08/11/19) and number of ESTs derived from these clusters are shown [41]. No NBL ESTs were members of the clusters derived from these genes.
(DOCX)
