# Supplementary figures and images for: Vaccination with novel low-molecular weight proteins secreted from Trichinella spiralis inhibits establishment of infection
Source: PLoS Negl Trop Dis. 2020 Nov 18;14(11):e0008842. doi: 10.1371/journal.pntd.0008842 (PMC7673540; doi:10.1371/journal.pntd.0008842)

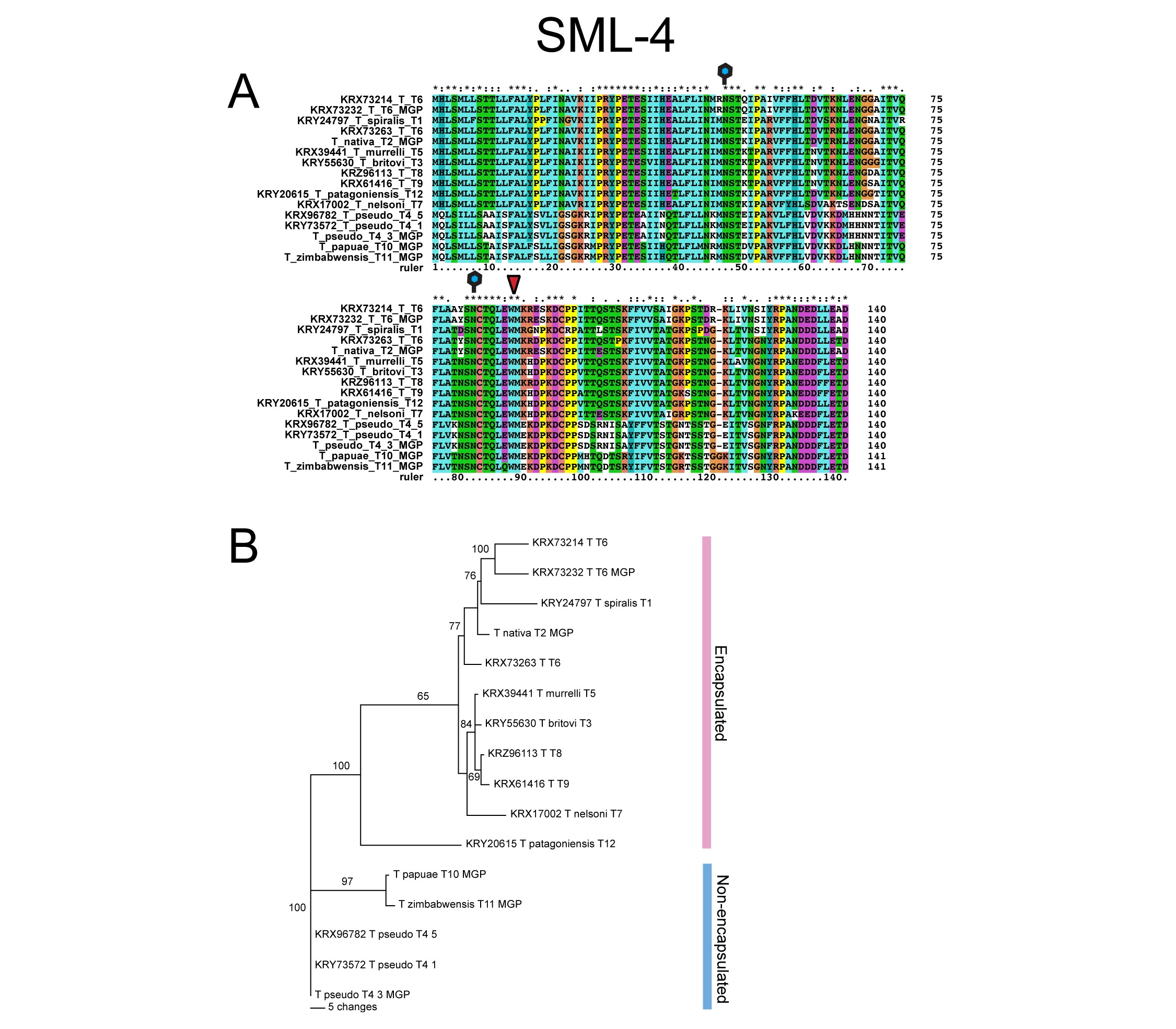

Supplement: S1 Fig — (A) A multiple protein sequence alignment yielded by ClustalX analysis of the 16 SML-4 homologues found in sequenced genomes of Trichinellid species contained in Genbank. The Genbank accession number of the protein is given along with the species/isolate identifier. In five instances a manual genome prediction (MGP) was required to refine gene prediction models obtained from searching contigs isolated from the T. nativa (T2, JYDW01000232.1), Trichinella (T6, JYDK01000182.1), T. pseudospiralis (T4_3, JYDV01000046.1), T. papuae (T10, JYDO01000115.1) and T. zimbabwensis (T11, JYDP01000179.1) genomes. The location of the conserved intron site is indicated with a red triangle and potential N-glycosylation sites with the blue hexagons. (B) A rooted cladogram showing the consensus of the tree found in the maximum parsimony analysis of the SML-4 protein sequence alignment. Nodes which are supported bootstrap values of > 60% are shown and the bootstrap values are placed at the base of the node. (TIF) [file pntd.0008842.s001.tif]

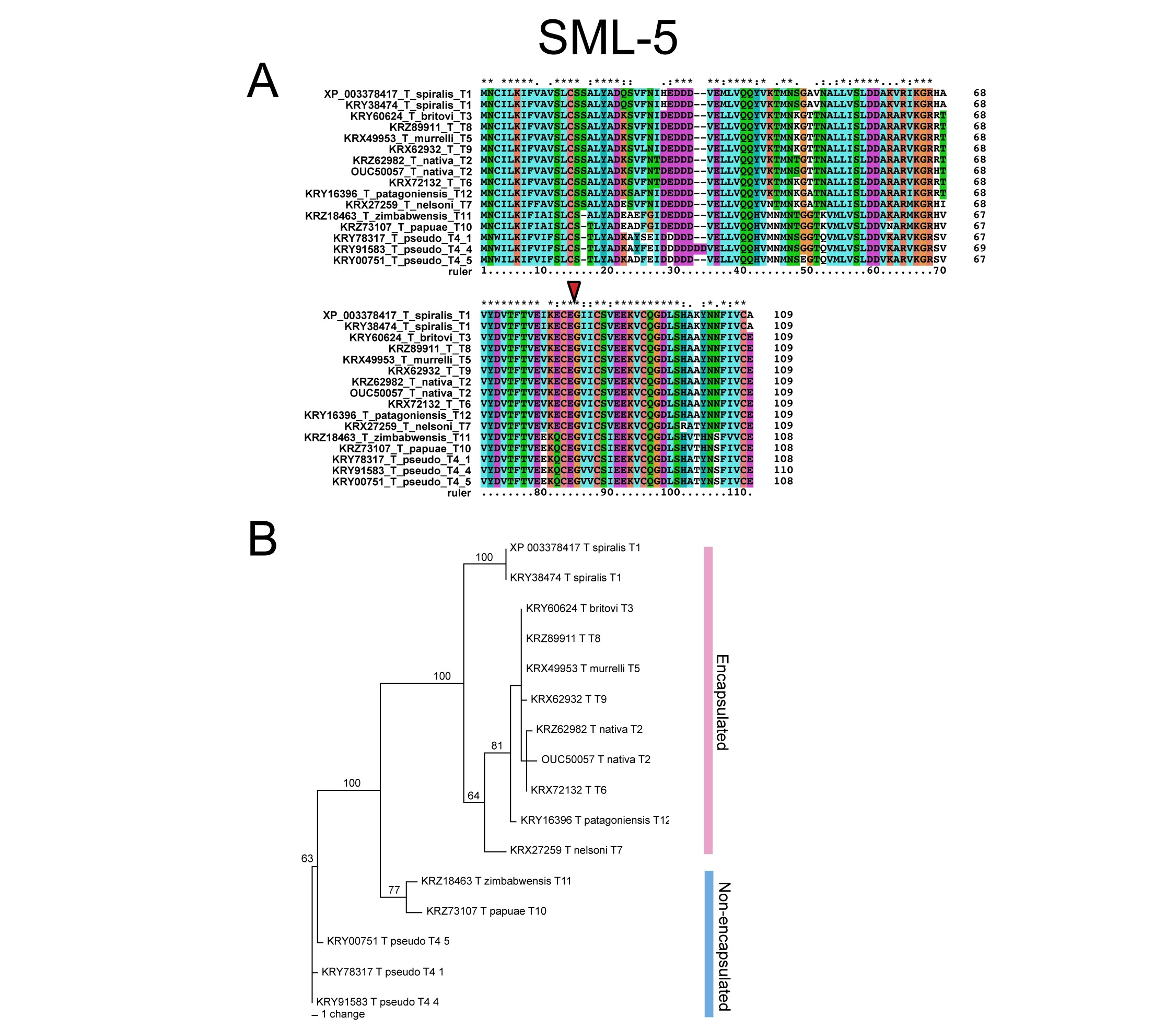

Supplement: S2 Fig — (A) A multiple protein sequence alignment yielded by ClustalX analysis of the 16 SML-5 homologues found in sequenced genomes of Trichinellid species contained in Genbank. The Genbank accession number of the protein is given along with the species/isolate identifier. The location of the conserved intron site is indicated with a red triangle. (B) A rooted cladogram showing the consensus of the tree found in the maximum parsimony analysis of the SML-5 protein sequence alignment. Nodes which are supported bootstrap values of > 60% are shown and the bootstrap values are placed at the base of the node. (TIF) [file pntd.0008842.s002.tif]

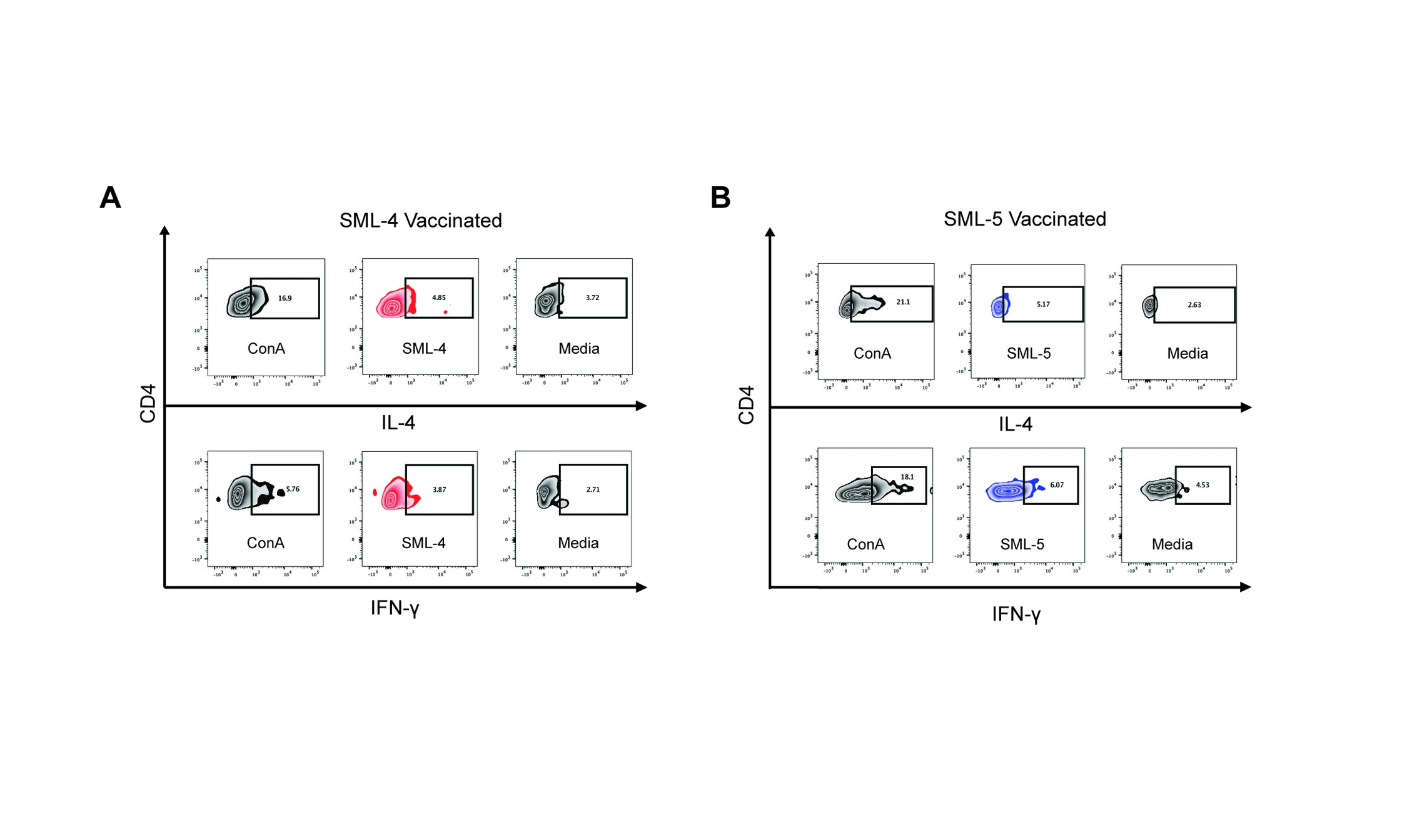

Supplement: S3 Fig — Flow cytometry of activated CD4+ cells following recall responses in BALB/c mice vaccinated with SML-4 (A) or SML-5 (B) protein in alum adjuvant. Cell stimulations (ConA, SML-4, SML-5, or media) are indicated within respective flow plots. Figures are representative of two independent experiments with n = 5 per group. (TIF) [file pntd.0008842.s003.tif]
