# Supplementary material for: Vaccination with novel low-molecular weight proteins secreted from Trichinella spiralis inhibits establishment of infection
Source: PLoS Negl Trop Dis. 2020 Nov 18;14(11):e0008842. doi: 10.1371/journal.pntd.0008842 (PMC7673540; doi:10.1371/journal.pntd.0008842)
Supplement: S1 Table — This table shows the top 11 candidates identified in the informatics screen of the T. spiralis mL1 EST dataset with their names and Genbank accession numbers. The columns next to the identifier show the number of ESTs for each transcript that were identified in the T. spiralis mL1 and adult EST datasets. Homologies to sequences contained within Genbank, the NEMBASE4 cluster (accessed 08/11/19) and number of ESTs derived from these clusters are shown [41]. No NBL ESTs were members of the clusters derived from these genes. (DOCX) [file pntd.0008842.s004.docx]

| **Name/ Identifier** | **mL1** | **Adult** | **Homologies and EST cluster numbers** |
| --- | --- | --- | --- |
| GRN-1/ EU867517.1 | 40 | - | Granulin domains (TSC00180, TSC10692 and TSC12476) [1] |
| SML-1 / EU867515.1 | 47 | - | Novel (TSC00585, TSC08102, TSC01706, TSC10742 ) [1] |
| gp45/ U01847 | 48 | 21 | similar to serine proteinases (TSC00054, TSC07448, TSC00054, TSC03042, TSC12723) [2] |
| 9.10/ MCD-1/ SML-2/ DQ777102 | 70 | 61 | Contains 3 domains with weak similarity to type II cystatins (TSC07780, TSC00403, TSC11698, TSC14929, TSC12815 TSC01154 and 32 additional clusters) [1,3] |
| 11.3/ SML-3/ EU867516 | 24 | 10 | Novel (TSC00935, TSC00437) [1] |
| SML-4/ KRY36588.1 | 46 | 1 | Novel (TSC00495, TSC03642, TSC08176, TSC07871, TSC06474) |
| Ts53/ U25127 | 79 | 3 | Weak similarity to nudix hydrolases. (TSC00078, TSC01684 [4] |
| gp43/ M95499 | 28 | - | Similar to DNAse II. (TSC07731,  TSC00010, TSC02314) [5] |
| prosaposin/ AY485648 | 61 | - | Similar to saposins. (TSC00370, TSC001230, TSC08772, TSC02110, TSC10677, TSC001369, TSC12160) [6] |
| GM2A/ DQ132801 | 18 | - | Contains lipid interacting domain and similarity to GM2 activator. (TSC00821) [7] |
| SML-5/ XP_003378417.1 | 12 | - | Novel (TSC00154, TSC14627) |

**S1 Table. Candidates isolated from *T. spiralis* mL1 EST datasets.**

This table shows the top 11 candidates identified in the informatics screen of the *T. spiralis* mL1 EST dataset with their names and Genbank accession numbers. The columns next to the identifier show the number of ESTs for each transcript that were identified in the *T. spiralis* mL1 and adult EST datasets. Homologies to sequences contained within Genbank, the NEMBASE4 cluster (accessed 08/11/19) and number of ESTs derived from these clusters are shown [8]. No NBL ESTs were members of the clusters derived from these genes.

**Reference:**

1. Guiliano DB, Oksov Y, Lustigman S, Gounaris K, Selkirk ME. Characterisation of novel protein families secreted by muscle stage larvae of Trichinella spiralis. Int J Parasitol. 2009;39(5):515-24. Epub 2008/11/11. doi: 10.1016/j.ijpara.2008.09.012. PubMed PMID: 18992250; PubMed Central PMCID: PMCPMC2680962.

2. Arasu P, Ellis LA, Iglesias R, Ubeira FM, Appleton JA. Molecular analysis of antigens targeted by protective antibodies in rapid expulsion of Trichinella spiralis. Mol Biochem Parasitol. 1994;65(2):201-11. Epub 1994/06/01. doi: 10.1016/0166-6851(94)90072-8. PubMed PMID: 7526209.

3. Robinson MW, Massie DH, Connolly B. Secretion and processing of a novel multi-domain cystatin-like protein by intracellular stages of Trichinella spiralis. Mol Biochem Parasitol. 2007;151(1):9-17. Epub 2006/11/04. doi: 10.1016/j.molbiopara.2006.09.008. PubMed PMID: 17081632.

4. Zarlenga DS, Gamble HR. Molecular cloning and expression of an immunodominant 53-kDa excretory-secretory antigen from Trichinella spiralis muscle larvae. Mol Biochem Parasitol. 1990;42(2):165-74. Epub 1990/09/01. doi: 10.1016/0166-6851(90)90159-j. PubMed PMID: 2270099.

5. Vassilatis DK, Despommier D, Misek DE, Polvere RI, Gold AM, Van der Ploeg LH. Analysis of a 43-kDa glycoprotein from the intracellular parasitic nematode Trichinella spiralis. J Biol Chem. 1992;267(26):18459-65. Epub 1992/09/15. PubMed PMID: 1382055.

6. Selkirk ME, Hussein AS, Chambers AE, Goulding D, Gares MP, Vasquez-Lopez C, et al. Trichinella spiralis secretes a homologue of prosaposin. Mol Biochem Parasitol. 2004;135(1):49-56. Epub 2004/08/04. doi: 10.1016/j.molbiopara.2004.01.005. PubMed PMID: 15287586.

7. Bruce AF, Gounaris K. Characterisation of a secreted N-acetyl-beta-hexosaminidase from Trichinella spiralis. Mol Biochem Parasitol. 2006;145(1):84-93. Epub 2005/10/26. doi: 10.1016/j.molbiopara.2005.09.010. PubMed PMID: 16242793.

8. Elsworth B, Wasmuth J, Blaxter M. NEMBASE4: the nematode transcriptome resource. Int J Parasitol. 2011;41(8):881-94. Epub 2011/05/10. doi: 10.1016/j.ijpara.2011.03.009. PubMed PMID: 21550347.
